# Reconstruction of acquired ischiatic and perineal defects: an anatomical and clinical comparison between gluteal thigh and inferior gluteal perforator flaps

**DOI:** 10.1007/s00238-017-1371-2

**Published:** 2017-11-25

**Authors:** Eduardo Montag, Thiago Ueda, Alberto Okada, Bruno Onishi, Rolf Gemperli

**Affiliations:** 1 Cancer Institute of São Paulo (ICESP), University of São Paulo, Alameda Campinas, 977 3 floor, São Paulo, Brazil; 2 Division of Plastic Surgery, University of São Paulo School of Medicine, São Paulo, Brazil

**Keywords:** Pressure ulcer, Surgical flaps, Comparative anatomy, Gluteal region, Thigh anatomy

## Abstract

**Background:**

Flap coverage is the gold standard in treating pressure sores, and due to the high recurrence rate, the possibility of multiple surgical procedures should be considered during flap selection. The gluteal thigh (GT) flap has become a workhorse for ischiatic pressure sore treatment at our hospital. Follow-up revealed a group of patients presenting recurrence of the pressure sore that needed a second flap. The inferior gluteal artery perforator (IGAP) flap was chosen in this series. The positive experience with both flaps raised the question of which flap should be the first option for the treatment of ischiatic and perineal pressure sores.

**Methods:**

IGAP and GT flaps were dissected in 21 fresh human cadavers to allow comparison of anatomical features. In a series of 60 patients, the authors used both the gluteal thigh and the IGAP flap to cover 76 ischiatic and perineal ulcers.

**Results:**

The IGAP flap was found to be wider and thicker than the gluteal thigh, but presented a shorter pedicle. All flaps healed uneventfully. Recurrent ulcers were treated successfully with both flaps.

**Conclusions:**

Both flaps are suitable for coverage ischiatic and perineal sores. Due to its anatomical features, the IGAP flap should be considered the first choice of treatment for ischiatic ulcers. The gluteal thigh flap should be used in the recurrent sores.

Level of Evidence: Level IV, therapeutic study.

## Introduction

Treatment of pressure sores (PS) is a challenge for plastic surgeons due to the tendency for recurrence [1]. Surgical management of the PS is controversial. Differences of opinion exist about what type of reconstruction should be done. However, for full thickness pressure sores, surgery remains the best option. Indeed, conservative treatment increases the possibility for early recurrence as healing by secondary intention usually results in unstable scars [2]. In these cases, both myocutaneous and fasciocutaneous flaps have been used successfully. These flaps provide bulk eliminating dead space, adequate blood supply to overlying soft tissues, and resistance to infections [3].

The gluteal thigh (GT) flap was described by Hurwitz in 1980 for perineal and ischiatic reconstructions. It is a fasciocutaneous flap based on the descending branch of the inferior gluteal artery. Described more recently, the IGAP flap is a perforator flap based on perforating branches from the inferior gluteal artery. The main advantages are derived from the reduced donor site morbidity. In our experience, both flaps are reliable and predictable, representing adequate choices for ischiatic and perineal reconstructions.

The reconstructive surgeon is constantly challenged to investigate and develop techniques to improve functional and esthetic outcomes. Utilizing the IGAP and GT flaps for ischiatic and perineal defects, reconstruction provides appropriate functional outcome while preserving local muscle function. Although reconstruction with local flaps is a well-described procedure and there are previous series evaluating the surgical results [4–7], there are few reports comparing the anatomy of GT and IGAP flaps [8, 9]. Moreover, less information is available concerning the surgical planning outcome of large wound reconstruction with these flaps. Thus, the purpose of this article is to describe the main anatomical parameters in cadavers to establish landmarks to flap dissection and report our clinical experience focusing attention on preoperative planning, outcome, advantages, and limitations of both techniques.

## Material and methods

### Anatomical study

**Fig. 1:**
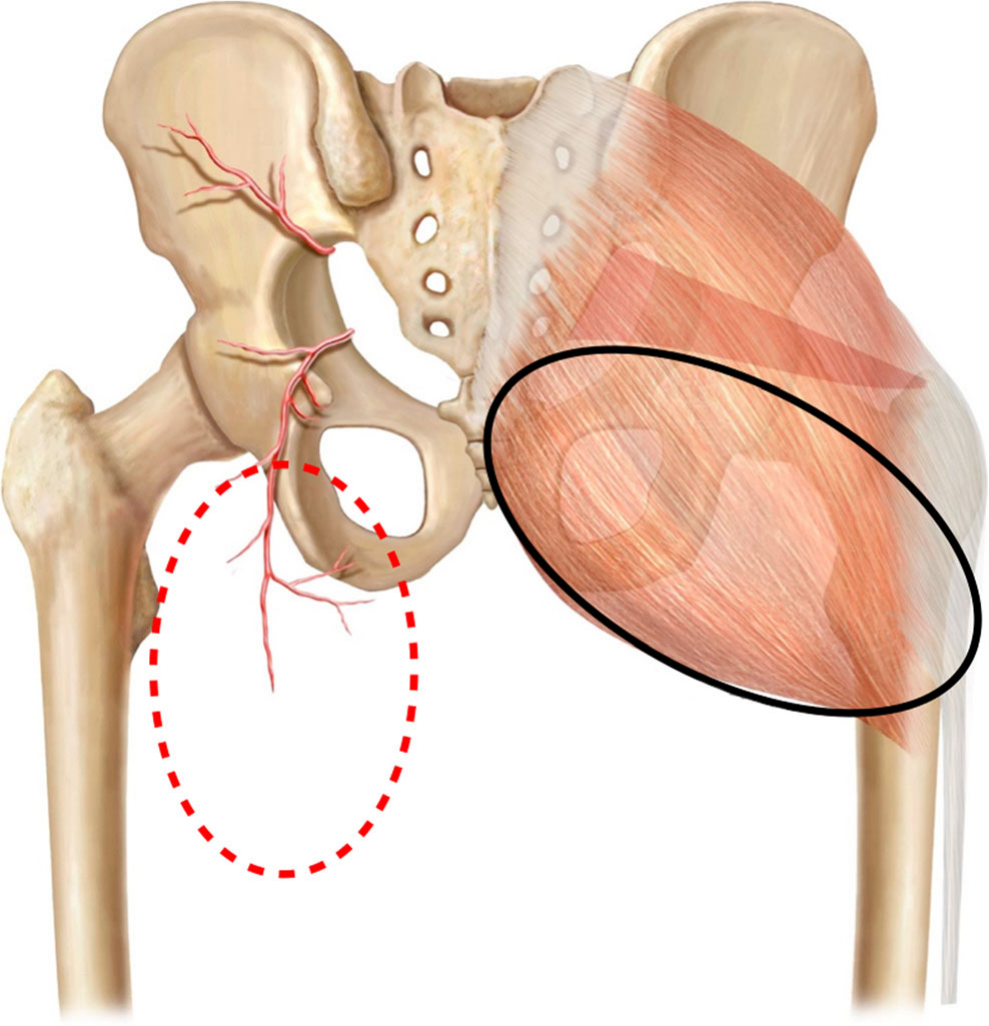
Location of both flaps and their relation to the underlying anatomy. GT flap-red. IGAP flap-black. The inferior gluteal artery can be seen as well as some perforators and the descending branch

**Fig. 2:**
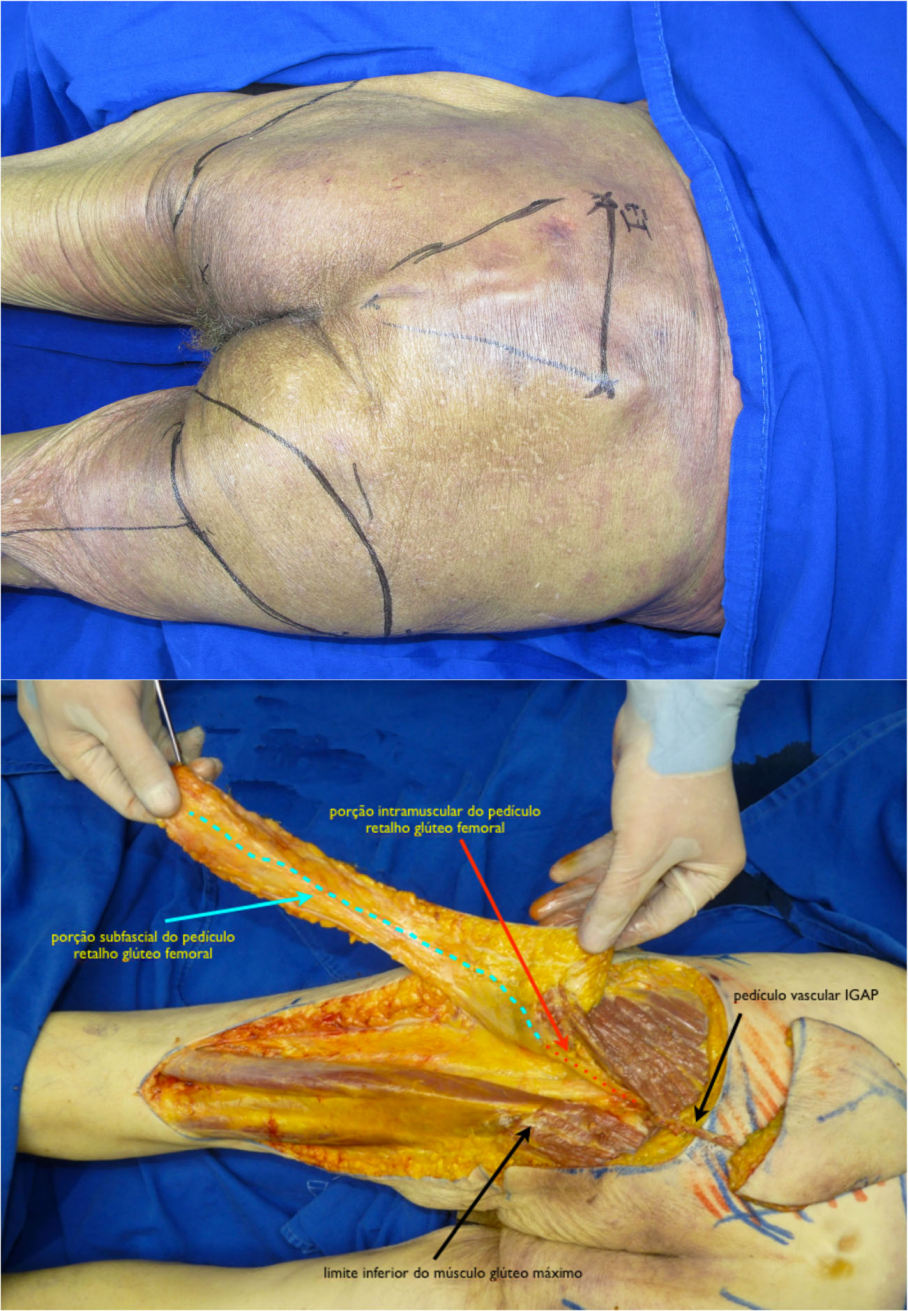
Preop markings (up). Dissection of both flaps showing the relation between the vascular pedicles. Red dots—intramuscular portion of GT pedicle. Blue dots—subfascial portion of GT pedicle. Black arrows—IGAP vascular pedicle (right); inferior border of gluteus maximus muscle (left)

Thirty-seven anatomical dissections of the gluteal and thigh region on 21 fresh cadavers (less than 24 h after death) were performed. Mean age was 53.8 years (sd 5.7), weight 67.5 kg (sd ± 7.2), and height 1.69 m (sd ± 0.05). Cadavers with previous gluteal and thigh surgeries were excluded. In order to determine the main anatomical characteristics of both flaps, the dimensions (width/length), thickness, and pedicle length were evaluated separately (Figs. 1 and 2).

#### Gluteal thigh flap

**Fig. 3:**
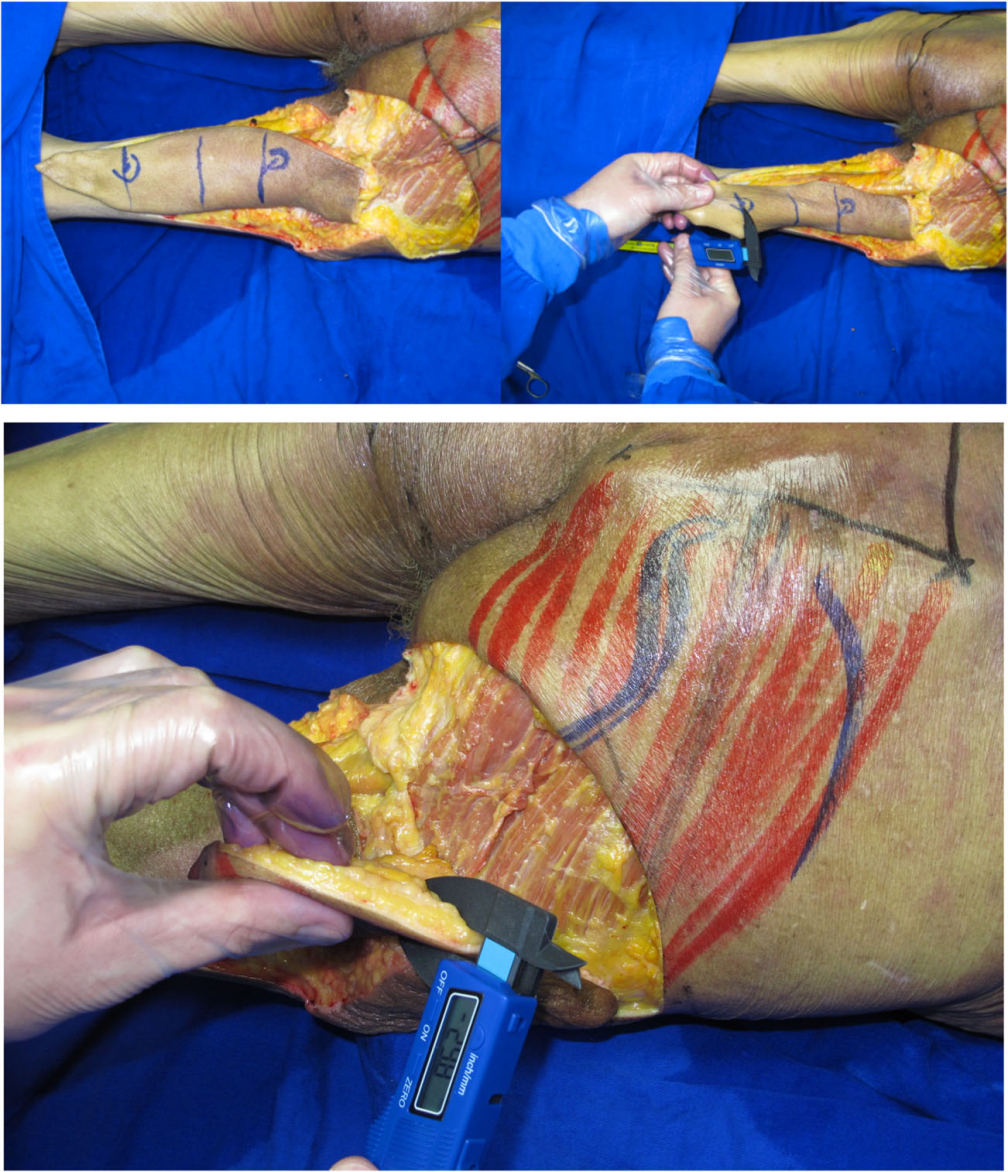
Thickness measures in both flaps

With the cadaver in the prone position, a point located in the mean distance between the ischial tuberosity and the greater trochanter was marked at the level of the gluteal crease. A line was drawn from this point to the popliteal crease in order to locate the vascular pedicle. The lateral and medial limits were determined through a pinch test. The flap was dissected in the subfascial plane, with direct visualization of the pedicle until its origin in the inferior gluteal artery. The following parameters were studied:

flap dimensions (width, length, and thickness); length of the vascular pedicle (Fig. 3).

#### Inferior gluteal artery perforator flap

The anatomical landmarks for the IGAP flap are the ischial tuberosity medially, the greater trochanter laterally, and the inferior gluteal crease inferiorly. The superior edge of the flap was obtained through a pinch test. Dissection was performed in the subfascial plane and the perforators were identified. The most suitable perforator was selected based on location and external diameter. The pedicle was then dissected down to its origin at the inferior gluteal artery. Care was taken to preserve the descending branch of the inferior gluteal artery. The following parameters were studied: flap dimensions (width, length, and thickness); length of the vascular pedicle.

### Clinical study

**Fig. 4:**
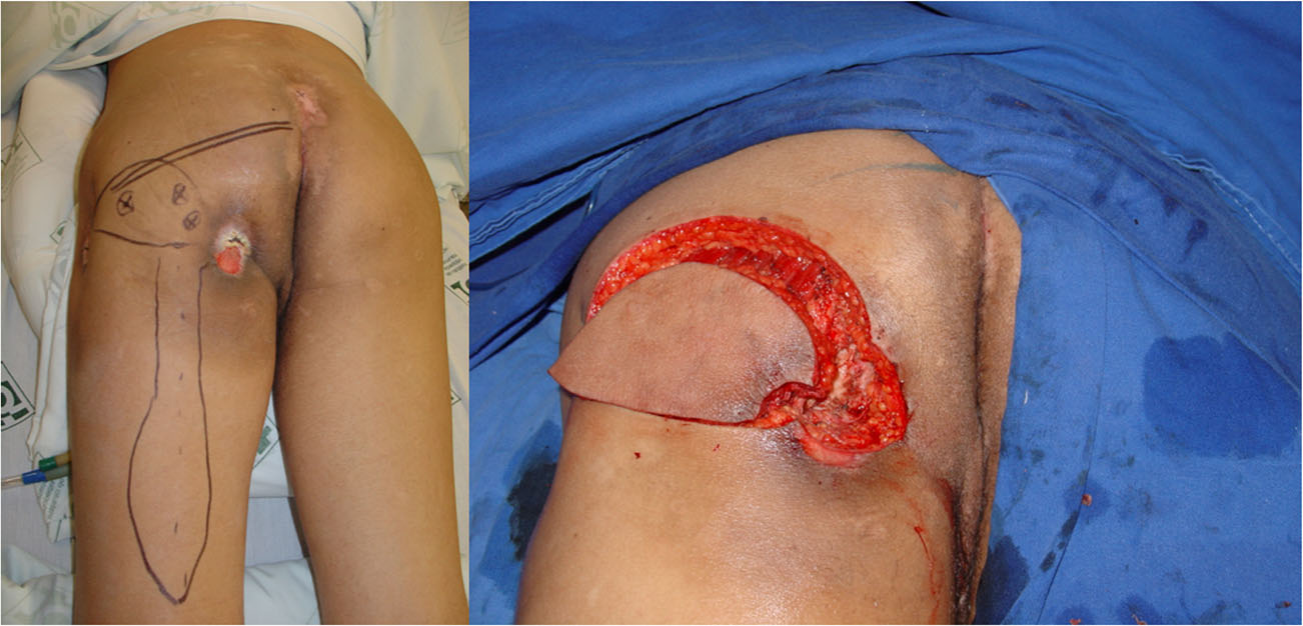
Recurrent ischiatic pressure sore. IGAP and GT flaps outlined (left). IGAP flap margins incised after wound debridement

Between January 2008 and December 2015, all cases submitted to ischiatic and perineal reconstruction at the University of São Paulo Medical School (HC-FMUSP) and the senior author’s (E. M.) private practice were reviewed. During this period, patients presenting PS and submitted to GT and IGAP flap reconstruction were selected. Clinical information on defect size and location, associated clinical diseases, and previous surgeries was obtained. Complications were evaluated and included skin necrosis, wound dehiscence, partial and total flap loss, infection, seroma, hematoma, and recurrence (Fig. 4).

#### Patient evaluation and reconstructive procedure

**Fig. 5:**
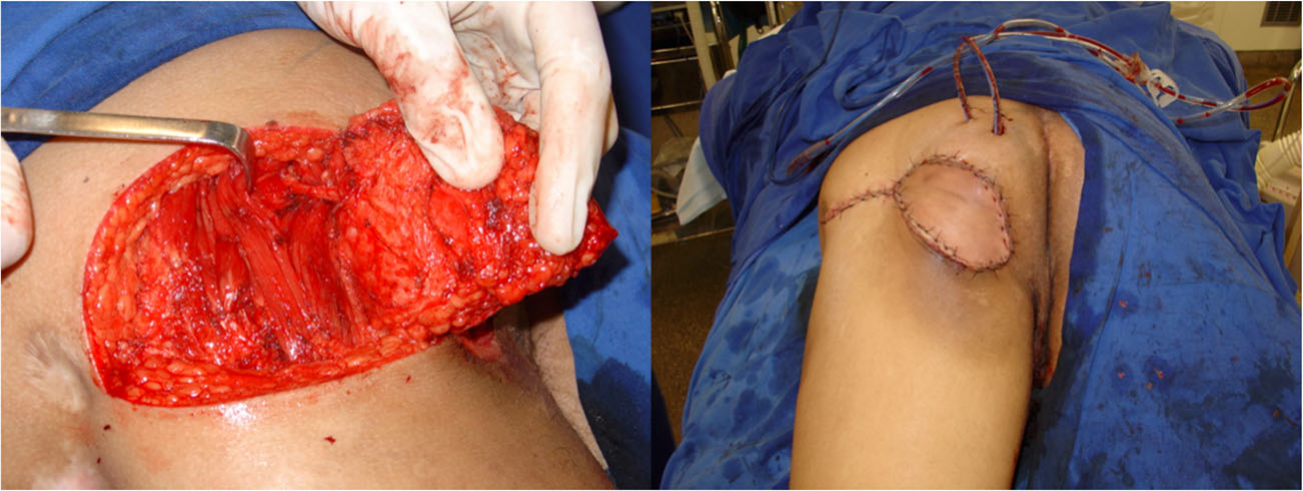
Elevated IGAP flap with vascular pedicle isolated (left). Immediate postop (right)

All patients were first seen by a multidisciplinary team. After adequate clinical treatment and compliance to the surgical protocol, the patient was scheduled for surgery. First, the wound is debrided and radical bursectomy is performed. Bone removal is performed only when necrosis or infection is suspected. Flap coverage providing filling of dead space and adequate padding is performed. The choice of flap for reconstruction depends on the location, size, and depth of the ulcer (Fig. 5).

#### The gluteal thigh flap

##### Planning/surgical technique

**Fig. 6:**
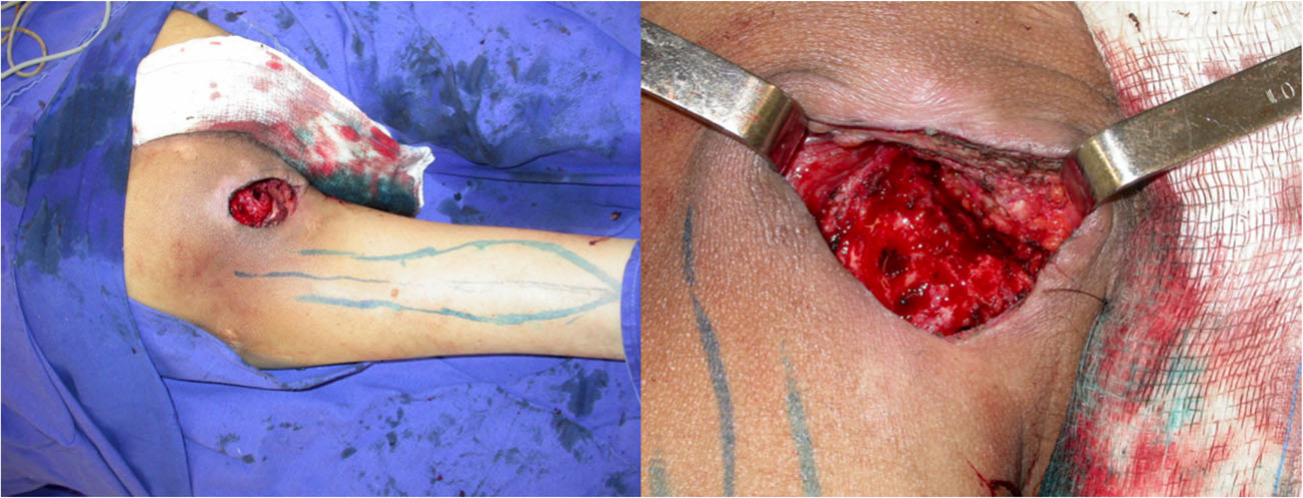
Ischiatic pressure sore. Gluteal thing flap outlined (left). Bone stump debridement detail (right)

Preoperative evaluation was done with the patient in prone position. The flap presents a rectangular shape with the base located at inferior gluteal crease. The skin incisions are made and electrocautery is used to divide the flap down to the posterior muscles of the thigh. The posterior femoral cutaneous nerve is identified at the distal part of the flap. The vascular pedicle runs parallel to the nerve deep to the fascia. Dissection proceeds in the subfascial plane from distal to proximal until the pivot point, normally located at the level of the gluteal crease, is reached. None of the cases required gluteal maximus muscle division (Fig. 6).

#### The inferior gluteal perforator flap

##### Planning/surgical technique

**Fig. 7:**
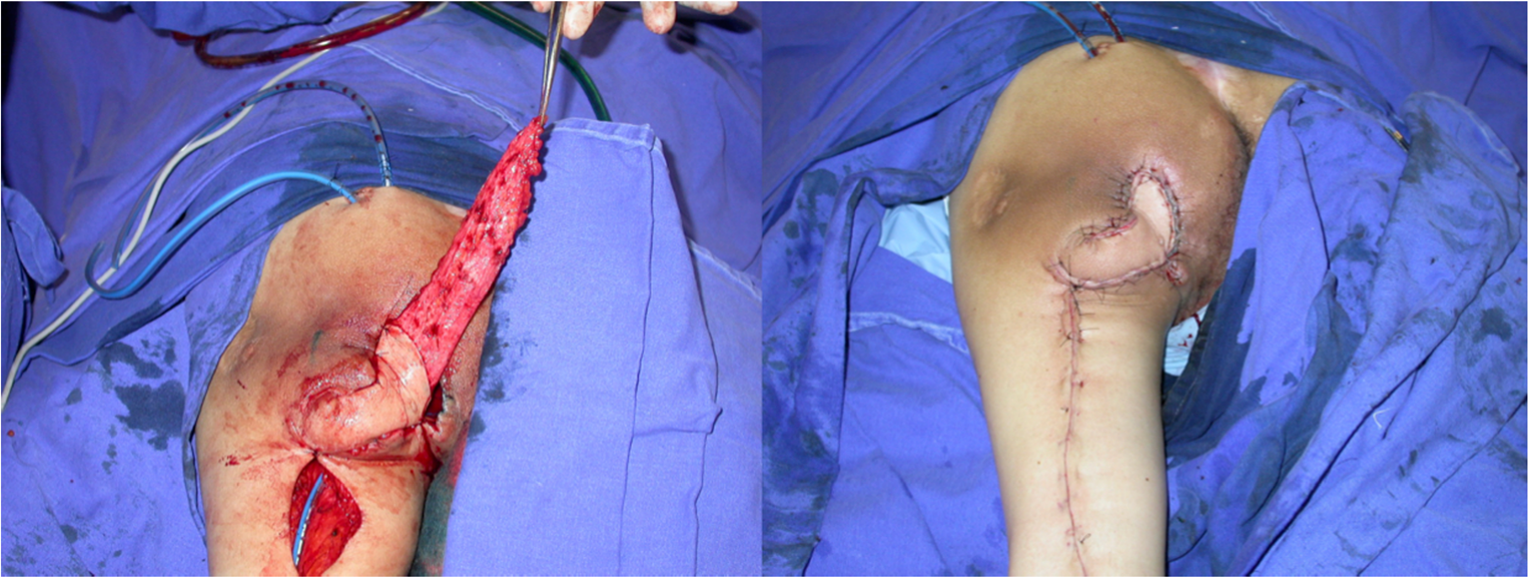
Flap rotated to defect with de epithelialized tip to fill dead space (left). Immediate postoperative (right)

Preoperative evaluation was done with the patient in prone position. The flap presents a fusiform shape with the inferior limit being the inferior gluteal crease. Usually, the skin paddle is marked in an oblique pattern from inferior medial to superior lateral to include the main perforators. The inferior gluteal artery is a terminal branch of the internal iliac artery and leaves the pelvis through the greater sciatic foramen inferior to the piriformis muscle. The artery is accompanied by the greater sciatic nerve, the internal pudendal vessels, and the posterior femoral cutaneous nerve. The skin incisions are made and electrocautery is used to divide the flap down to the muscle of the gluteus maximus. The flap is elevated from the muscle in the subfascial plane and the perforators approached from lateral to medial. After a suitable perforator is chosen, intramuscular dissection is carried out until adequate pedicle length allows for flap mobilization. Care is taken to preserve the descending branch of the inferior gluteal artery in case the patient develops a new pressure sore in the future (Fig. 7).

## Results

### Anatomical study

The results are summed up in Table 1.

**Table 1:** The IGAP flap was found to be thicker and wider than the gluteal thigh flap. On the other hand, the gluteal thigh flap has a longer pedicle, as well as a bigger length of the skin island

	IGAP		Gluteal thigh	
	Mean	Standard deviation	Mean	Standard deviation
Width (cm)	18.06	1.93	8	3.6
Length (cm)	12.03	1.84	20	3
Thickness (cm)	2.38	0.61		
Proximal thickness (cm)			2.06	0.6
Distal thickness (cm)			1.66	0.42
Number of perforators	15.09	2.6		
Number of large perforators	4.67	0.42		
Number of small perforators	10.42	2.48		
Pedicle length (cm)	7.78	0.93	23.12	7.5

### Clinical study

Sixty patients with ischiatic or perineal pressure sores were included in the study. Group 1 comprised of 25 patients treated prior to the anatomical study. All the patients received a gluteal thigh flap. All the flaps healed well with only minor complications. Eight of the patients presented recurrence and needed an additional flap. IGAP was chosen as alternative with success in all the cases. Dissection was difficult in some patients because of the scar tissue. After the anatomical study, the indication of the flap was made based on the characteristics of the lesion such as size, depth, and location. Thirty-five patients were treated in the second group. Twenty-five patients received an IGAP flap and 10 a GT flap. Total complication rate was 18%. All complications were considered minor ones and treated conservatively. There were 4 wound dehiscence (donor area), 3 superficial infections, 2 fistulas, and 2 seromas. No recurrences were observed in group 2 patients after an 18-month follow-up.

## Discussion

Pressure sore surgery remains a challenge for plastic surgeons due to the tendency for recurrence. The reported prevalence is high as 26% among hospitalized patients and 39% among patients with spinal cord injuries [10, 11]. Additionally, the complications and the recurrence rates are the major problems following surgical treatment, which are previously described variedly from 7 to 62% [12, 13].

Many options are available for surgical management of PS, including direct closure, skin grafting, fasciocutaneous flaps, and musculocutaneous flaps. Immediate postoperative complications and ulcer recurrence rates at follow-up have been remarkably high, particularly in patients with spinal cord injuries. In spite of these limitations, these high incidences can be reduced by comprehensive care provided by the multidisciplinary team. With adequate knowledge of surgical techniques and particularities of each region to be treated, a satisfactory long-term outcome can be obtained.

While treating ischiatic and perineal defects, both fasciocutaneous and muscle flaps can be selected. The decision to use a particular flap depends on the surgeon’s expertise and on patient and ulcer characteristics. Many flaps have been described to treat PS in these regions. Muscle flaps such as the hamstring, gracilis, and the gluteus maximus have been used in the spinal cord injury population with success. For ambulatory patients, muscle flaps should be avoided to preserve function. Traditionally, the myocutaneous flap has been described as the first choice, by eliminating the dead space, providing adequate blood supply to overlying soft tissues, and superior resistance to infections [3]. However, Thiessen et al. in a large study of 94 PS reconstructions utilizing myocutaneous and fasciocutaneous flaps observed that complication and recurrence rates were not associated with the type of the flap [14].

Gluteal flaps have been largely utilized in reconstructive surgery [15]. However, flap dissection is difficult, and exposure of the donor vessels risks injury to the adjacent sciatic nerve. In addition, partial resection of the gluteus maximus muscle results in weakness of thigh abduction and extension in ambulatory patients. Recently, the IGAP flap has been used to repair pressure sores [16]. This flap can be harvested without significant damage to associated muscles, thereby reducing the postoperative morbidity. However, the variable anatomy and the necessity for intramuscular dissection of perforators have been described as the main limitations of the procedure. In recent years, perforator flaps have been widely used in reconstructive surgery becoming the gold standard in many areas.

The major advantage of fasciocutaneous and perforator flaps is the preservation of the underlying muscle, which is particularly important to ambulatory patients and patients with a high tendency for recurrence. In case of a recurrence, these flaps can be readvanced as random flaps. In terms of perineal defects, a thin flap is the ideal alternative. It requires a simple and safe procedure with minimal invasion and preserves the underlying donor tissues for further reconstruction. For the ischiatic region, depending on the depth of the defect, more bulk is required.

The anatomical study showed that the IGAP flap is thicker than the gluteal thigh flap in both its proximal and distal portions. This fact can be explained by the distribution of the fat in the body. While in the gluteal region the subcutaneous tissue is thick, in the posterior thigh region, the thickness decreases from proximal to distal. The indication of the IGAP flap as the first option to cover ischiatic pressure sores can be done based on the like with like concept. When selecting the gluteal thigh flap, the thickness can be increased through deepitelialization and folding of the distal portion of the flap [17].

Flap dimensions obtained through the pinch test showed that the IGAP has a bigger cutaneous island when compared to the gluteal thigh flap (216 cm 2 × 160 cm^2^). The IGAP is a wide flap while the gluteal thigh has its width limited by skin elasticity of the thigh region. On the other hand, the length of the gluteal thigh is bigger than that of the IGAP flap. As a consequence, each flap should be indicated for a certain type of defect. In clinical practice, both of the flaps are suitable for the treatment of most of the defects located in the ischial and perineal areas. The IGAP flap has the advantage of multiple designs of the skin island while the gluteal thigh has an almost fixed design.

Comparison between the vascular pedicle of the flaps is difficult due to the distinct anatomical features. The gluteal thigh flap is a fasciocutaneous flap based on the descending branch of the inferior gluteal artery. The pedicle has a short intramuscular course and a long fasciocutaneous course. The IGAP flap is based on a perforator pedicle with origin in the inferior gluteal artery and a long intramuscular course. The differences become clear when the length of the pedicles is compared. The gluteal thigh has a longer pedicle (23.12 cm) and the IGAP has a shorter pedicle (7.78 cm). In our paper, the length of the IGAP flap pedicle is shorter than previously described because the dissection was not continued to the origin in the inferior gluteal artery to preserve the descending branch [18].

We would like to acknowledge some limitations of the paper. It is an anatomical study performed in fresh cadavers, which facilitates the dissection and measures when compared with the ones done in preserved cadavers. It is possible that the dimensions of the skin islands obtained through the pinch test do not correspond to the real size observed during dissection of the flaps. Clinical application has proved both flaps to be safe and larger than observed during the dissections as previously demonstrated by many authors [19–25].

## Conclusion

IGAP flap is thicker and has a shorter pedicle than the gluteal thigh flap. These features and the fact that the IGAP flap is closer and similar to the tissues lost to the pressure sore make it the first choice to treat ischiatic pressure sores. The gluteal thigh flap, due to the reduced thickness, longer pedicle, and wider arc of rotation, should be regarded as an option on recurrent sores or in patients presenting ulcers located in the medial parts of the perineum.
